# Genome-wide identification of cancer/testis genes and their association with prognosis in a pan-cancer analysis

**DOI:** 10.18632/oncotarget.21715

**Published:** 2017-10-10

**Authors:** Vandeclecio Lira da Silva, André Faustino Fonseca, Marbella Fonseca, Thayna Emilia da Silva, Ana Carolina Coelho, José Eduardo Kroll, Jorge Estefano Santana de Souza, Beatriz Stransky, Gustavo Antonio de Souza, Sandro José de Souza

**Affiliations:** ^1^ Instituto do Cérebro, UFRN, Natal, Brazil; ^2^ Ph.D. Program in Bioinformatics, UFRN, Natal, Brazil; ^3^ Bioinformatics Multidisciplinary Environment (BioME), Digital Metropolis Institute, UFRN, Natal, Brazil; ^4^ Instituto de Bioinformática e Biotecnologia, Natal, Brazil; ^5^ Instituto Metrópole Digital, UFRN, Natal, Brazil; ^6^ Departmento de Engenharia Biomédica, UFRN, Natal, Brazil

**Keywords:** cancer/testis, cancer antigens, biomarkers, prognosis

## Abstract

Cancer/testis (CT) genes are excellent candidates for cancer immunotherapies because of their restrict expression in normal tissues and the capacity to elicit an immune response when expressed in tumor cells. In this study, we provide a genome-wide screen for CT genes with the identification of 745 putative CT genes. Comparison with a set of known CT genes shows that 201 new CT genes were identified. Integration of gene expression and clinical data led us to identify dozens of CT genes associated with either good or poor prognosis. For the CT genes related to good prognosis, we show that there is a direct relationship between CT gene expression and a signal for CD8+ cells infiltration for some tumor types, especially melanoma.

## INTRODUCTION

Genes with a restricted pattern of expression in normal tissues and expression in tumors cells are excellent candidates for biomarkers and therapeutic targets. Among these genes, cancer/testis (CT) genes are the most promising with several clinical trials under way [1]. These CT genes are exclusive or predominantly expressed in testis among normal tissues but are also expressed in a variety of tumor types [2]. Few authors have suggested that these genes may be considered as cancer-germline (CG) genes, as they can also present regular expression in ovary and placenta [3, 4]. When protein products of CT genes elicit an immune response, they are called CT antigens [3]. Methodologies, like SEREX and protein arrays, have identified several CT genes as CT antigens. Cellular and humoral immune responses have been observed for many CT antigens in patients bearing a variety of tumor types [5–7], making them good candidate targets for cancer immunotherapy, like cancer vaccination, adoptive T-cell transfer with chimeric T-cell receptors or in combination with conventional cytotoxic therapies [8–12].

Although there is a lack of consensual classification of CT genes, authors have divided them into two distinct groups [13]. The first one is organized in multigene families generally located on the X chromosome, where they comprise approximately 10% of the genes on that chromosome (CT-X genes) and show heterogeneous expression in cancer tissues that increase during tumor evolution and can elicit immune responses in cancer patients. The other group comprises the non-X CT genes since they are located on autosomal chromosomes. This last class does not appear to exist as multigene families and are often expressed during meiosis [14]. In 2009, data from about 70 families of cancer testis antigen, with more than 200 members, were gathered into a database (http://www.cta.lncc.br/). This CT Database provides information about CT genes including names and aliases, genomic location among others, but has not incorporated data from new technologies and contains less than 250 CT genes [15]. Moreover, several other genome-wide analyses have been reported. Hofmann et al. [14], for example, used a combination of CAGE, MPSS and RT-PCR to perform a survey of cancer/testis genes. They identified more than 30 candidate CT genes. More recently, our group characterized the human surfaceome and identified 14 putative CT genes coding for cell surface proteins [16]. Using public microarray data, two genes FMR1NB and TMEM31 were characterized as CT genes coding for cell surface proteins, which render them excellent candidates for targeted therapies [16]. One of them, FMR1NB, was found to elicit immune response in sarcoma patients [17]. While the project reported here was underway, Wang et al. [5] reported a systematic identification of CT genes in 19 tumor types.

The development of next-generation sequencing (NGS) technologies catalyzed a series of projects whose primary objective was to genetically characterize a cohort of cancer patients and associate that information with clinical data. The most successful of these projects was “The Cancer Genome Atlas” (TCGA) that integrated information for more than 11,000 patients from a variety of tumor types. NGS technologies have also allowed a better characterization of the human transcriptome derived from healthy cells and tissues. Projects like the Human Body Map (HBM) (GEO accession: GSE30611), the Genotype-Tissue Expression (GTEx) [18] and the Human Protein Atlas (HPA) [19] have deep sequenced the human transcriptome of dozens of cell types.

While the genome-wide screens performed so far were necessary for a better characterization of the universe of CT genes, most of them were executed at a time in which these NGS technologies were not widely available. Yao [20] have recently used TCGA data to explore CT genes. They, however, restricted their analysis to a subset of known CT genes. On the other hand, Wang et al. [5] used TCGA data to perform a genome-wide screen for CT genes. In the present paper, we have integrated information derived from both HBM/GTEx/HPA and TCGA to provide a complete genome survey of CT genes with the characterization of 201 new putative CT genes. By using mass-spectrometry data, it is shown that several of our putative CT genes exist at the protein level in some tumor types. Finally, CT genes associated with either a good or poor outcome are identified. For some CT genes associated to good prognosis, an association to CD8+ cells infiltration is observed. We propose that CT genes whose expression is related to a good outcome are excellent candidates for immunotherapeutic approaches.

## RESULTS

### Identification of genes predominantly (or exclusively) expressed in testis

**Figure 1:**
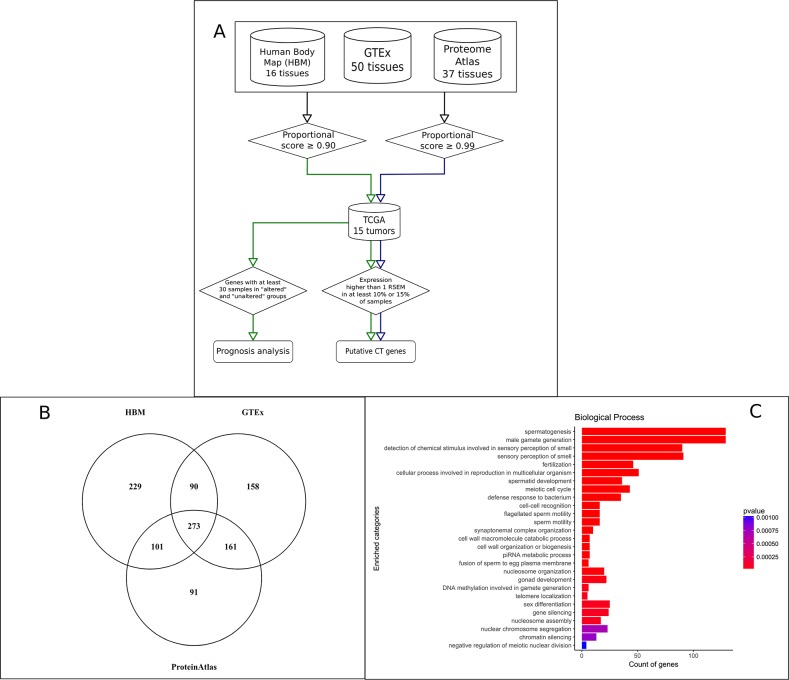
Identification of putative CT genes in 15 tumor types **(A)** Schematic view of the pipeline used to identify CT genes with the identification of 1,103 genes predominant or exclusively expressed in testis and 745 putative CT genes. **(B)** Venn diagram showing the intersection of the 1103 genes predominant or exclusively expressed in testis regarding their source. **(C)** Gene Ontology enrichment analysis using the set of 1,103 genes predominant or exclusively expressed in testis.

Assuming that CT genes are predominant or exclusively expressed in testis when compared to the remaining tissues, we used RNA-Seq data from three different sources to identify genes with expression bias to the testis. Absolute transcript level in each tissue was converted to a proportional score (transcript level in a tissue divided by the sum of levels in all tissues), and a threshold of at least 0.9 was used to select genes preferentially or exclusively expressed in testis. A threshold of 0.9 would imply that at least 90% of all expression of that gene in all analyzed tissues was derived from testis. HBM (GEO accession: GSE30611), GTEx [18] and the HPA [19] datasets, all reporting RNA-Seq data from a variety of normal tissues, were used to select genes preferentially (or exclusively) expressed in testis. The resulting selection, detailed in Figure 1A, contained a set of 1,103 non-redundant genes based on the three sources used in our analysis (Figure 1B and Supplementary Table 1 for a complete list of testis-biased genes). A gene ontology analysis in this gene set showed, as expected, a strong bias towards biological processes related to germline cells (Figure 1C).

To make available a set of putative CT genes identified using more stringent criteria, we modified our pipeline to select genes with a proportion score of at least 0.99. This selection generated a list of 793 genes with an exclusive expression in testis. While we have used the set of 1,103 genes in the remaining analysis, one can identify the more restricted set of testis-specific genes in Supplementary Table 1.

### Identification of putative CT genes

**Figure 2:**
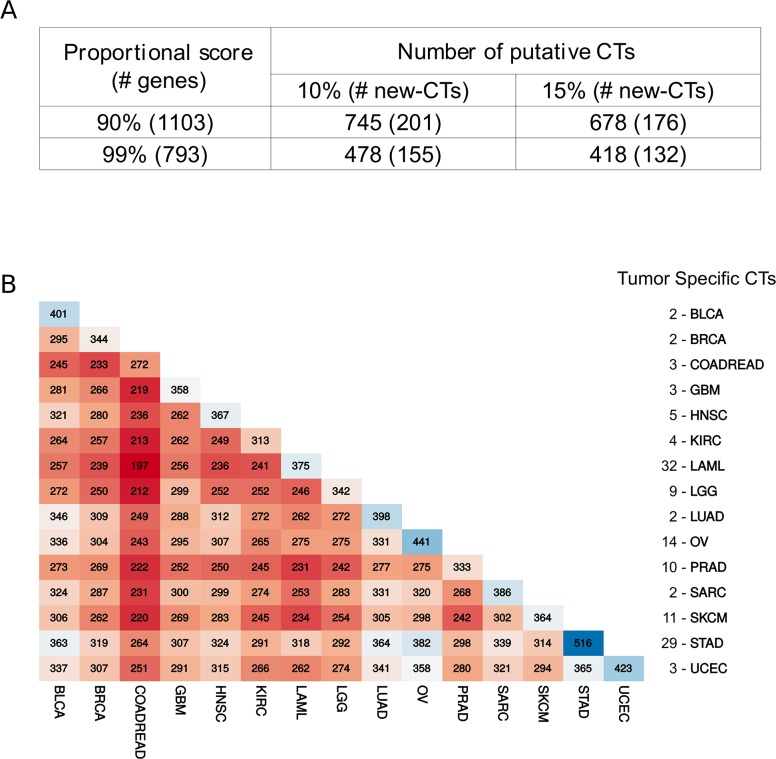
Comparative analysis of putative CT genes **(A)** Number of testis-biased genes and putative CT genes according to different stringent criteria for selection. **(B)** Matrix reporting the number of shared CT genes for all possible paired tumor type. The list at the right is the number of exclusive CT genes per tumor.

The expression pattern in tumors of the 1,103 genes predominantly expressed in testis was evaluated using the TCGA dataset. RNA-Seq data from 6,221 tumor samples were collected from the TCGA data repository comprising 15 tumor types. To identify genes significantly expressed in a given tumor, we used statistics provided by TCGA itself. A gene was considered a putative CT gene if it had a level of expression (cutoff threshold of RSEM >1) in at least 10% of all informative samples for a given tumor. These two criteria identified 745 putative CT genes (201 as new CT genes by comparing to the CT Database and data from Wang et al. [5]) significantly expressed in at least one tumor type (Figure 2A). If a more stringent threshold is used (15% of all samples for a given tumor type), we found 678 putative CT genes (176 as new CT genes) (Figure 2A). The same procedure was applied to the list of testis-biased genes identified using the more stringent 0.99 proportional score giving 478 (155 new) and 418 (132 new) putative CT genes using the 10% and 15% thresholds, respectively (Figure 2A). Supplementary Table 2 lists all genes with their corresponding expression in all analyzed tumor types. A comparative analysis between all tumor types, shown in Figure 2B, reveals that among the putative CT genes one can find several that are tumor-specific. In several cases, a particular CT gene showed a significant expression in only one tumor (17% of all CT genes). For example, 32 CT genes were expressed exclusively in leukemia, 11 in melanoma, and 14 in ovarian cancer (Figure 2B). This finding demonstrated the potential of our strategy for proposing particular biomarkers and targets for different types of tumors.

### CT genes as cancer genes?

Much has been discussed on the role of CT genes in driving tumorigenesis [12]. Based on that, we decided to explore our set of putative CT genes regarding their status as cancer genes in the TCGA dataset used here. A method recently developed by us, the S-score [21], integrates information such as mutation screening, methylation status, copy-number variation and expression profiling and was used to infer whether our CT candidates showed a pattern of either an oncogene or tumor suppressor. The S-score was calculated for all 1,103 genes preferentially expressed in testis for all 15 tumor types studied here. When a stringent cut-off was used, as in De Souza et al. [21] (S-score >3, indicating an oncogene, or <-3, showing a tumor suppressor), we found 313 cancer genes (128 oncogenes and 201 tumor suppressors) among the 1,103 genes preferentially expressed in testis. Few genes behave differently in distinct tumor types. Supplementary Table 2 lists the S-score values for all testis-biased genes in all 15 tumor types used here.

**Figure 3:**
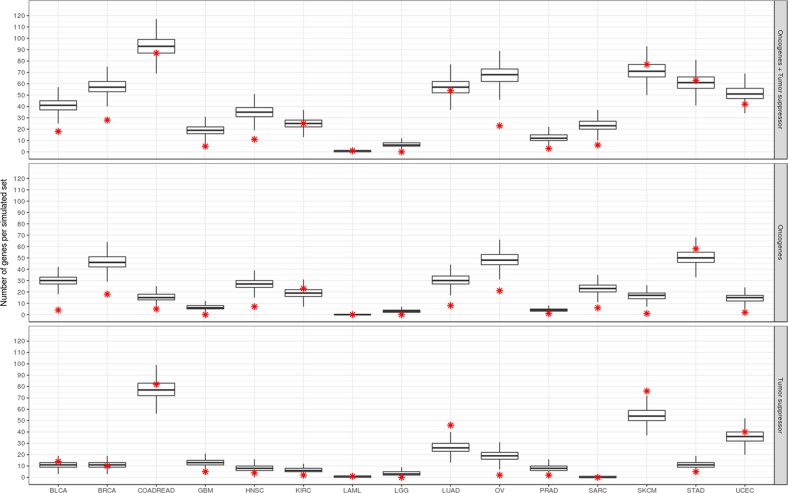
Enrichment analysis of CT genes for oncogenes and tumor suppressors Box plots represent the distribution of the number of cancer genes in the 10,000 Monte Carlo simulations. Red star indicates the true number of cancer genes in the set of CT genes for each respective tumor type. Upper, middle and lower panels correspond to the real and simulated sets for oncogenes and suppressors together, oncogenes and suppressors, respectively.

To evaluate whether the set of putative CT genes is enriched or depleted of cancer genes in any tumor type, a Monte Carlo simulation was performed using random sets of 1,103 genes. As shown in Figure 3, there is an overall depletion of oncogenes in this set of testis-biased genes (pattern found in 12 out of 15 tumor types). Only five tumor types showed a depletion of suppressors while two tumor types showed an enrichment of suppressors (LUAD and SKCM). No oncogene enrichment was found. The same pattern, general depletion of cancer genes in the set of CT genes, was also observed when we used the set of 745 putative CT genes in the simulations (Supplementary Figure 1). This finding strongly suggests that, while there are several cancer genes within the CT genes dataset, there is overall a depletion of cancer genes in that set except for SKCM and LUAD, both presenting enrichment for tumor suppressors.

### Protein products of CT genes detected in tumor samples

To serve either as a biomarker or a therapeutic target, a given CT gene should be expressed at the protein level. Mass spectrometry-based strategies are becoming powerful resources to query the proteome, and the golden standards technologies are now able to identify around 10,000 proteins per sample. We capitalize on the availability of mass spectrometry data from different sources for a variety of tumor samples to determine among the putative CT genes those expressed at the protein level.

Using a cohort of 209 samples from different tumor types (04 melanoma, 95 colorectal tumors, 40 breast tumors, 36 prostate tumors and 34 ovary tumors), we identified 136 putative CT genes at the protein level (Supplementary Table 3). Although this is a significant number, it should be emphasized that a high rate of false-negatives is expected in this analysis due to the non-exhaustive nature of mass spectrometry-based approaches. By plotting the sum of the area under curve measurements of all identified proteins in each sample group (Supplementary Figure 2), it is possible to observe that many of these CT gene products belong to the top 50% of the most abundant proteins in the detectable proteome of the sample.

A comparison of the Top 15 most abundant CT genes in the proteomic dataset shows that some genes are more globally present in most or all tumors represented here, such as PBK, SPATA22, IL4I1, HIST1H1A, among other. Some genes seem to show a more specific profile depending on the tumor type, such as C17ORF104, only highly-abundant in the melanoma samples, FUT5 in colon, PAGE1 in ovarian tumor and CSNK1A1L in prostate.

### CT signature for cancer prognosis

**Figure 4:**
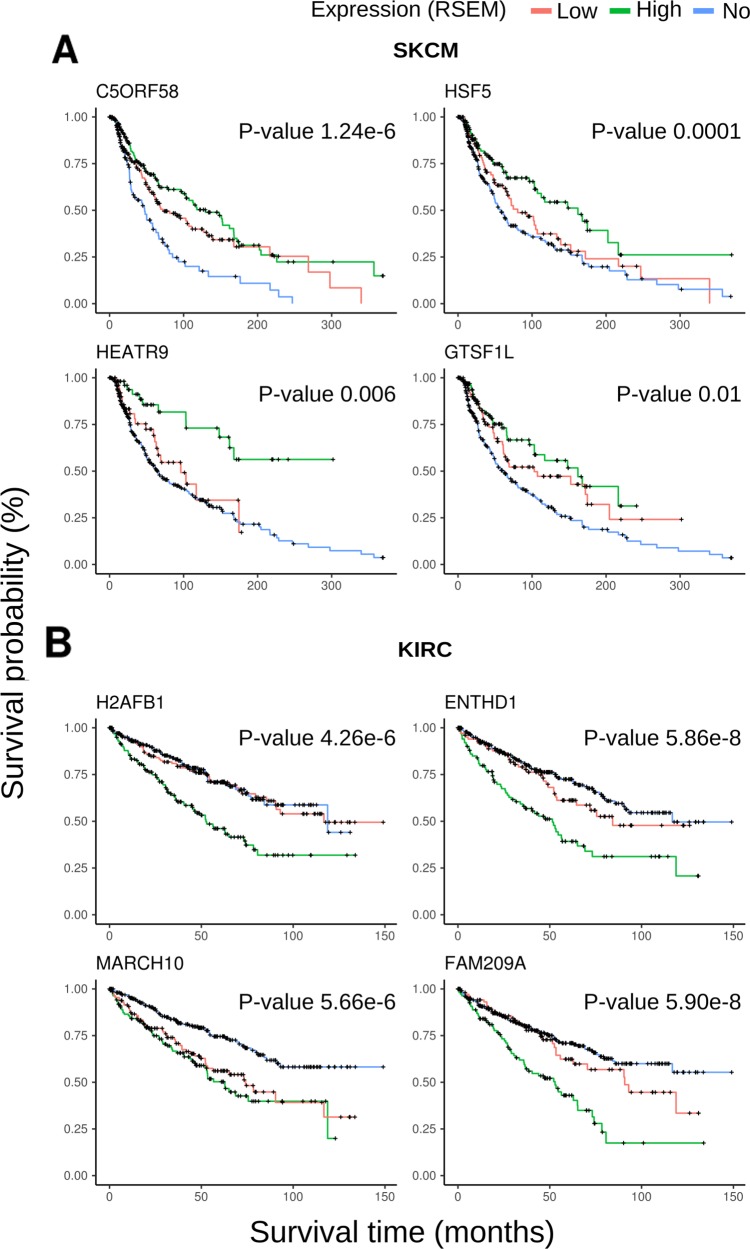
Kaplan-Meyer plots for representative CT genes **(A)** Most representative plots for SKCM including four genes associated to good prognosis. **(B)** Most representative plots for KIRC including four genes associated to poor prognosis.

Clinical data regarding overall survival from TCGA patients was used to evaluate the impact of expression of a putative CT gene in the outcome of the corresponding patients. A computer program evaluated all putative CT genes (745) for all 15 tumor types regarding any survival difference between samples expressing (FPKM or RSEM >1) or not expressing a given CT gene. Supplementary Table 4 provides the raw data for this survival analysis for all tumor types. Genes reporting a q-value < 0.05 (as defined by a log-rank test) for the difference in survival between the groups of patients expressing or not expressing a given CT gene were selected for further analysis. Overall we found 207 CT genes (non-redundant) whose expression affected the outcome of patients in at least one tumor type. Expression of CT genes was more associated with poor prognosis (179 genes) than to better prognosis (57 genes). Few genes behave differently in distinct tumor types. We confirmed the finding from Yao et al. [20] who found a high frequency of CT genes affecting prognosis in kidney renal clear cell carcinoma (KIRC). Most of the CT genes in KIRC were associated with poor prognosis (129 out of 141 genes). A more even distribution of CT genes associated to good and poor prognosis were found for LGG (30 and 32 CT genes, respectively) and SKCM (5 and 5 CT genes, respectively). To further select potential candidates, we split samples based on the expression of a given CT gene in three categories: no expression, expression below the median and expression above the median. If the expression of a corresponding CT gene was truly associated with patient outcome, we would expect that patients expressing more of the corresponding CT gene would have a stronger survival effect. A manual inspection of the Kaplan-Meyer plots for all 236 CT genes (179 and 57 associated to poor and good prognosis, respectively) was performed looking for the above pattern, and 113 genes were found more strongly associated with patient outcome (illustrative plots for KIRC and SKCM are shown in Figure 4).

**Figure 5:**
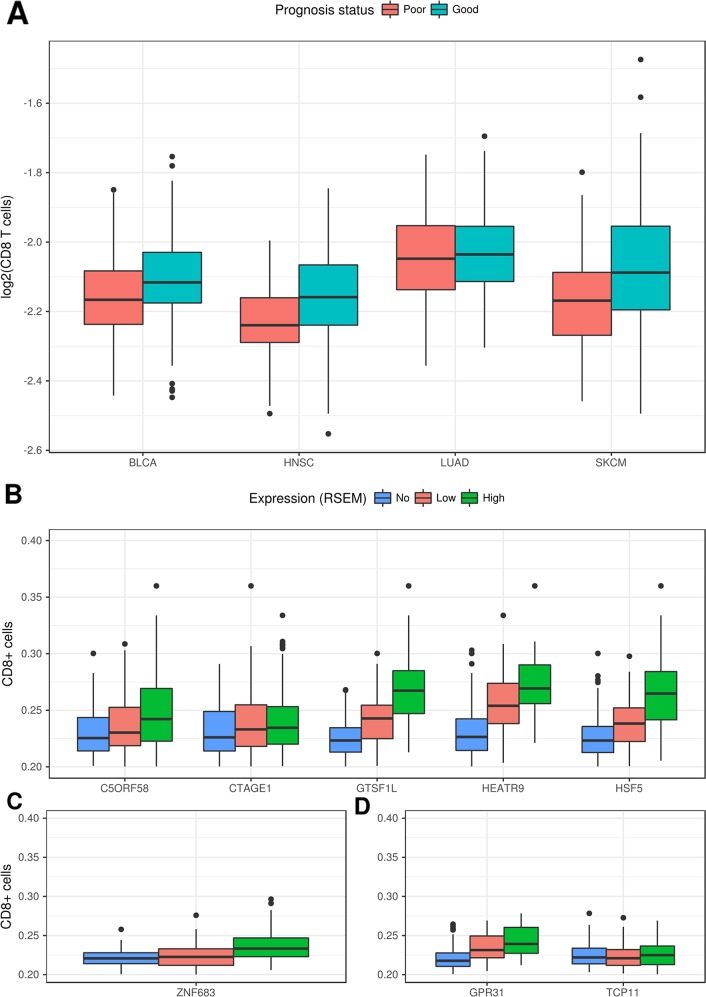
Correlation between expression of CT genes and the number of infiltrating CD8+ cells **(A)** Samples with higher expression for CT genes associated to good prognosis have a higher rate of infiltrating CD8+ cells for BLCA, HNSC and SKCM. **(B)** Rate of infiltrating CD8+ cells for samples with no, low or high expression for the respective CT genes associated to good prognosis in SKCM. **(C)** Rate of infiltrating CD8+ cells for samples with no, low or high expression for the respective CT genes associated to good prognosis in HNSC. **(D)** Rate of infiltrating CD8+ cells for samples with no, low or high expression for the respective CT gene associated to good prognosis in BLCA.

Data from Senbabaoglu et al. [22], who also used expression data from TCGA, was then used to assess the association between expression of a given CT gene and the number of infiltrating CD8+ cells. Senbabaoglu et al. [22] developed a method that uses expression data from TCGA to estimate the number of imune cells infiltration in a given tumor. Our analysis was only possible for four tumor types (BLCA, HNSC, LUAD and SKCM) due to either the lack of a significant number of CT genes associated with good prognosis or the lack of data of infiltrating CD8+ cells for some tumors. For two tumor types, LGG and KIRC, the high number of CT genes associated to both, good and poor prognosis, rendered the comparison impossible since both groups of samples were almost identical (since they comprised the totality of the samples). Figure 5A shows that samples with high expression of CT genes associated with good prognosis have a significantly higher number of CD8+ cells in BLCA (p < 9.9e^−5^), HNSC (p<9.5e^−4^) and SKCM (1.1e^−6^). When we split the samples according to the expression of a given CT gene, for most of the genes associated with good prognosis there is a significant association between the CT gene expression and infiltration of CD8+ cells for SKCM (Figure 5B), HNSC (Figure 5C) and BLCA (Figure 5D). A scatchard plot (showing the association between expression and CD8+ infiltration) for the same genes is shown in Supplementary Figure 3. Stronger associations were obtained for ZNF683 in BLCA, GPR31 in HNSC and C5ORF58, GTSF1L, HSF5 and HEATR9 in SKCM.

## DISCUSSION

We have capitalized on the availability of NGS data to perform one the largest screening for CT genes so far. One first critical issue, as discussed by [5] and [14], is the classification of CT genes based on its expression pattern in normal tissues. These genes exhibit different expression profiles and can be categorized into testis-restricted, testis/brain-restricted, or testis-selective group. This pattern imposes a challenge to identify the CT genes that are most suitable for the development of cancer therapies. Hofmann et al. [14], for example, performed an expression survey of 153 known CT genes and showed that only 39 had an exclusive expression in testis while the remaining had at least some expression in other tissues. Our screening of genes with a testis-biased expression involved the use of two proportional scores with different levels of stringency (0.9 and 0.99). While we used the less stringent dataset in most of the remaining analysis, the more stringent dataset is available to the community (Supplementary Table 1).

Several reports in the literature have indicated that CTs are mainly expressed in lung, ovarian, bladder, breast tumors and especially melanoma [3]. Most of the studies so far have used approaches based on the interrogation of few genes although Hofmann [14] used a series of high-throughput gene expression analyses to validate putative CT genes and Wang et al. [5] have used TCGA data from 19 tumor types. TCGA has provided a unique opportunity to screen for putative CT genes in a large panel of samples from many different tumor types and associated clinical features. Yao [20] have used TCGA data to explore the pan-cancer expression landscape of CT genes restricting their analysis to a subset of the CT database. Here, again, we used two thresholds with different levels of stringency (expression in at least 10% or 15% of samples in a given tumor) to identify putative CT genes. This allowed us to identify 745 putative CT genes (using the 10% threshold). By comparing this set of CT genes with the catalogs from the CT database and from Wang et al. [5] we found 201 new CT genes in our dataset. Several CT genes showed expression in only one tumor, which demonstrate the potential of our analysis for proposing biomarkers and targets for particular types of tumor.

The CT gene catalog generated by this study allowed us to evaluate some questions regarding CT genes. For example, much has been discussed on the role of CT genes in driving tumorigenesis [12]. One of the arguments that support a more important role of CT genes in tumorigenesis is related to their functions, many of which are related to tumorigeneses, like signal transduction and gene regulation. Another indirect support comes from an apparent similarity between germ cell and tumor developments [12, 23, 24]. In that line, some authors [25, 26] proposed that the expression of CT genes, usually restricted to germline cells, would trigger a gametogenic program in other somatic cells that would contribute to the tumorigenesis. Furthermore, some CT genes seem to be associated with the maintenance of an undifferentiated state of stem cells as reported by Cheng et al. [27] and Lifantsenva et al. [28]. Nevertheless, few CT genes have been shown to act as an oncogene (which would be expected based on their expression pattern). CT45A1 was shown to work as an oncogene and drive tumorigenesis in breast cancer [29]. Deletion of SSX2 in melanoma cells significantly reduced cell proliferation [30]. Members of the MAGE family have been implicated in cancer cell survival, frequently acting in the p53 pathway [31, 32]. Involvement with cancer progression, especially metastasis formation, has also been shown for some CT genes [29, 31, 33]. Our list of CT genes was evaluated for any potential enrichment of oncogenes and tumor suppressor, using the S-score method developed by us previously [21]. We found that in general there is a depletion of cancer genes in the set of CT genes. This finding strongly suggests that, while there are several cancer genes within the CT genes dataset, there is overall a depletion of cancer genes in that set except for melanoma and lung adenocarcinoma, both presenting enrichment for tumor suppressors.

Although few CT genes have been associated with either poor or good prognosis in a variety of tumor types [34–37], no large-scale analysis has been performed with a complete set of putative CT genes. Djureinovic et al. [38] generated a wide list of putative CT genes in non-small-cell lung cancer and found no gene associated to prognosis. Yao et al. [20] have recently shown that although some CT genes from the CT Database are associated with patient outcome, not many are independent prognostic markers. We explore this issue in a systematic way through an exhaustive analysis on the association between the expression of CT genes and cancer prognosis. Dozens of CT genes were associated with either good or poor prognosis. The robustness of our method is shown by the identification of genes clearly associated with disease progression among the set of 113 CT genes more strongly related to patient outcome by our analysis. Included in this set of genes are TEX101 [39], HORMAD2 [40], OTP [41] and TEX19 [42]. It is interesting to notice that CT genes are not significantly enriched for genes associated to prognosis (data not shown).

It is tempting to speculate that the CT genes associated to a better prognosis are eliciting an immune response against the tumor, which could be the reason for a better outcome in such patients. ROPN1 has been demonstrated to induce autoantibodies in patients with prostate cancer [43] and multiple myeloma [44]. Spontaneous tumor immune response was also detected for SPAG6 in sera from patients with gastric cancer, melanoma and prostate cancer [45]. CTAGE1 antibodies were also found in sera of colorectal cancer patients [46].

The finding that expression of several CT genes is associated with good prognosis led us to hypothesize that this effect could be the result of infiltrating CD8+ cells driven by the CT gene expression. Several methods have been developed that evaluate the intra-tumor immune landscape based on gene expression analysis of the bulk tumor [22, 47, 48]. Data from Senbabaoglu et al. [22] is quite suitable for our analysis since they used their method in most of the tumor types evaluated by us in this report. We found several CT genes, especially in SKCM, whose expression is significantly associated to both good prognosis and CD8+ cell infiltration. We suggest that these CT genes be considered for further studies that would evaluate their immunotherapeutic potential.

## MATERIALS AND METHODS

### RNA-Seq data source

Four datasets were used for the identification of CT genes, including three from normal tissues, obtained from the Expression Atlas Portal [18]: the Human Body Map (GEO accession: GSE30611), GTEx [18] and Human Protein Atlas (HPA) [19]. The 16 samples from the Human Body Map were processed using our pipeline [49]. Expression values for GTEx and HPA datasets were obtained directly from the projects web page as FPKM and TPM values, respectively. Data from 15 tumor types from the TCGA consortium was used for the identification of putative CT genes. Expression, methylation and GISTIC CNV data were obtained from the cBIO portal by using the CGDS-R package, which provides processed data for each tumor type. Furthermore, somatic mutation data from COSMIC [50] and a local compilation of all somatic mutations found in the literature [49, 50] were used. For each sample in each tumor type, an expression threshold equal to 1 RSEM was applied to separate samples based on the expression of the putative CTs. Only genes with expression in more than 10% or 15% of samples were considered CTs for a particular tumor.

### Enrichment ontology analysis

The R package “clusterProfiler” version 3.3 [51] was used to perform the ontology enrichment analysis based on Gene Ontology (GO) with a hyper-geometric test and correction method of Benjamini-Hochberg (BH), with cutoff parameters of p-value < 0.05 and q-value < 0.05. To remove redundancy of enriched GO terms the function *“simplify”* with default parameters was used.

### Proteomic analysis of public cancer datasets

The following MS raw file datasets were downloaded from ProteomeXchange: ovarian cancer dataset PXD003668 [52]; breast cancer dataset PXD002619 [53]; melanoma dataset PXD001724 [54]; colon cancer dataset PXD002041-50 [55]; prostate cancer dataset (PXD003430, PXD003452, PXD003515, PXD004132, PXD003615, PXD003636, PXD004159) [56]. All datasets were submitted to MaxQuant software version 1.5.2.8 [57] for protein identification. Parameters were set as follows: protein N-acetylation and methionine oxidation as variable modifications; carbamidomethylation of cysteine as fixed modification; first search error window of 20 ppm and main search error of 6 ppm at MS level. Furthermore, trypsin without proline restriction enzyme option was used, with two allowed mis-cleavages. Minimal unique peptides were set to 1, and FDR allowed was 0.01 (1%) for peptide and protein identification. The Uniprot human database was used (download from August 2016). Generation of reversed sequences was selected to assign FDR rates. A contaminants filter was performed, removing all occurrences presents on columns “Reverse” or “Potential contaminant” from the output of MaxQuant.

### S-score simulation

Identification of cancer genes was performed using the S-score metric [21] in both the set of testis-enriched genes (1103) and the set of 745 putative CTs. The Monte Carlo simulation was performed against each tumor type (with extreme S-score), where 10.000 simulated sets were compared to the real sets. In this step, three different tests were carried out: enrichment for oncogenes (genes with S-score ≥ 3), enrichment for tumor suppressor (genes with S-score ≤ -3) and enrichment for cancer genes (including both oncogenes and tumor suppressors).

### Survival signatures and patients prognosis

To test the association of CT genes with patient outcome in a given tumor type all putative CTs expressed in at least 30 samples were used. All putative CTs were tested individually using a log-rank test and genes were selected based on a threshold (q-value ≤ 0.05), as defined by the “qvalue” R package [58], and classified as associated with “Good” or “Poor” prognosis. Next, samples expressing a given CT associated with prognosis were separated in two subsets based on a median expression of the corresponding CT gene. Kaplan-Meyer curves were plotted using the ggplot2 (from the R package). CD8+ profiling for TCGA samples was obtained from Senbabaoglu et al. [22].

## SUPPLEMENTARY MATERIALS FIGURES AND TABLES











## Abbreviations

CT: Cancer/testis
CG: cancer-germline
NGS: next-generation sequencing
TCGA: The Cancer Genome Atlas
HBM: Human Body Map
GTEx: The Genotype-Tissue Expression
KIRC: kidney renal clear cell carcinoma
SKCM: skin cutaneous melanoma
GO: Gene Ontology
