# Supplementary material for: Genome-wide identification of cancer/testis genes and their association with prognosis in a pan-cancer analysis
Source: Oncotarget. 2017 Oct 10;8(54):92966–77. doi: 10.18632/oncotarget.21715 (PMC5696236; doi:10.18632/oncotarget.21715)
Supplement: Supplementary file 1 [file oncotarget-08-92966-s001.pdf]

Genome-wide identification of cancer/testis genes and their association with prognosis in a pan-cancer analysis

SUPPLEMENTARY MATERIALS

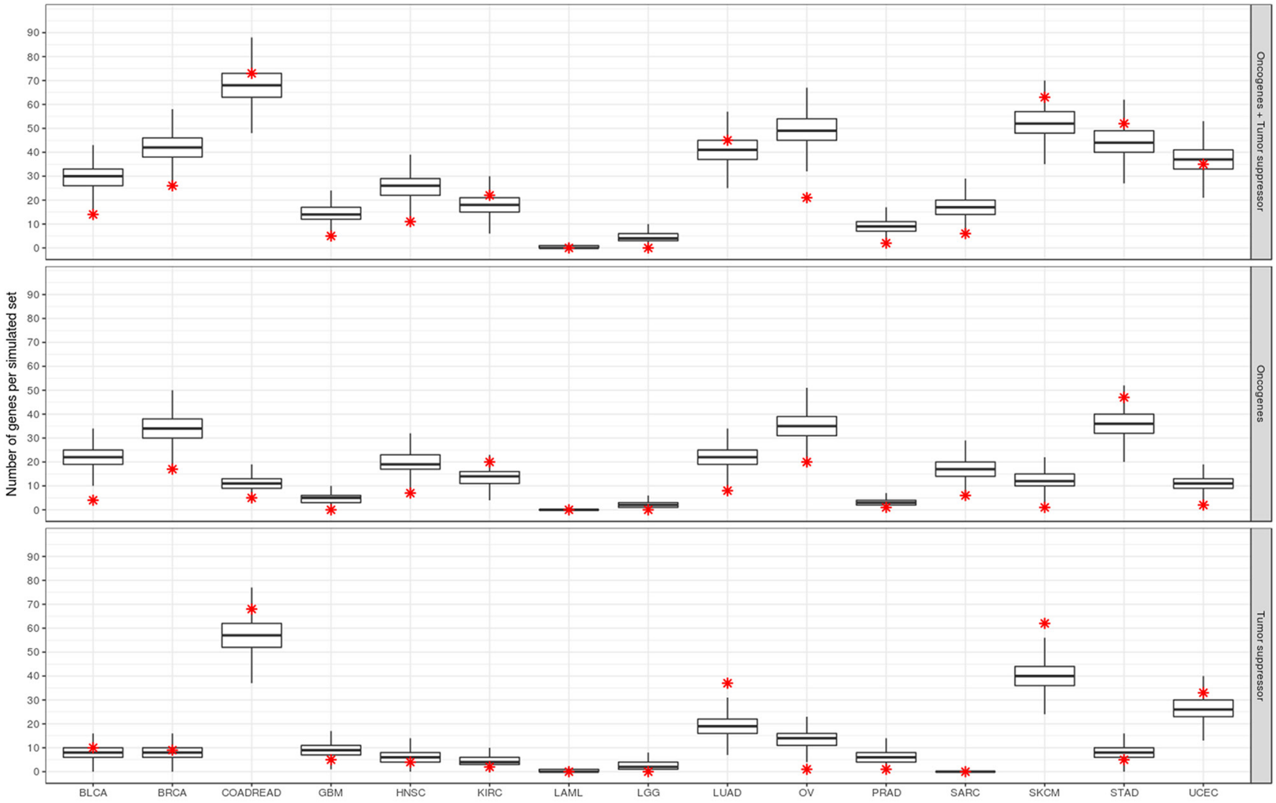

Supplementary Figure 1: Enrichment analysis of CT genes for oncogenes and tumor suppressors in the set of 745 putative CT genes. Box plots represent the distribution of the number of cancer genes in the 10,000 Monte Carlo simulations. Red star indicates the true number of cancer genes in the set of CT genes for each respective tumor type. Upper, middle and lower panels correspond to the real and simulated sets for oncogenes and suppressors together, oncogenes and suppressors, respectively.

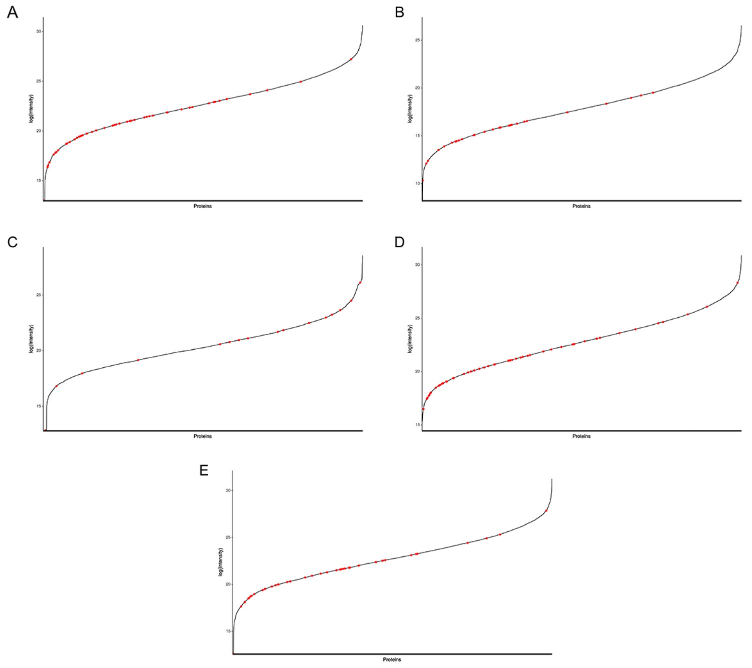

Supplementary Figure 2: Protein abundance plots. The area under curve quantitation as calculated by MaxQuant for each protein, per sample, was log2 transformed and plotted into a linear graph, from the least abundant (left) to the most abundant protein (right). Dots marked in red are protein products of CT genes identified in this work. **(A)** Breast, **(B)** colon, **(C)** melanoma, **(D)** ovarian, and **(E)** prostate.

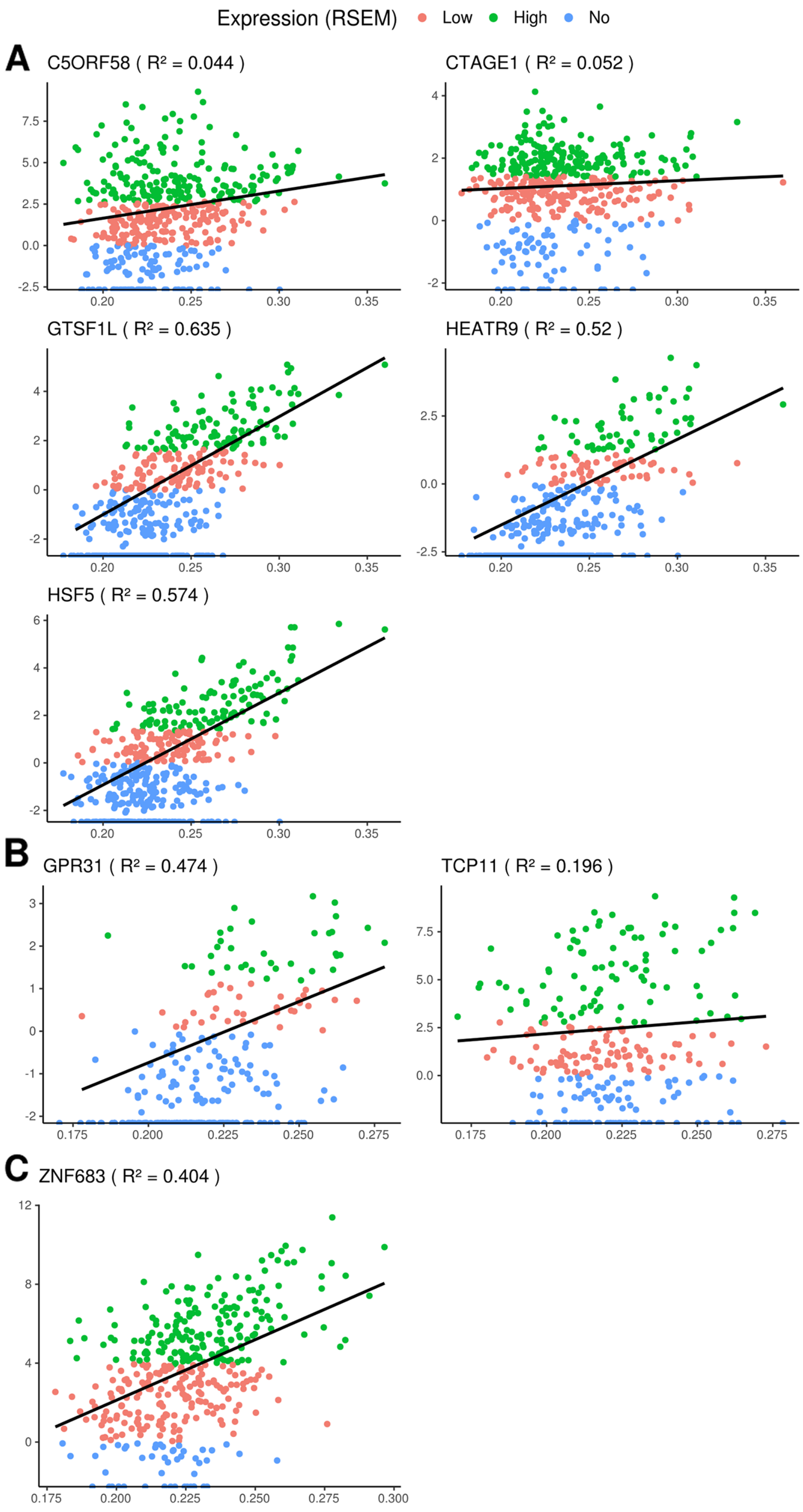

Supplementary Figure 3: Correlation between expression of CT genes and CD8+ count. Scatchard plots for the correlation between expression of CT genes and CD8+ count. **(A)** Plots for five CT genes associated to good prognosis in SKCM. **(B)** Plots for two CT genes associated to good prognosis in HNSC. **(C)** Plots for one CT gene associated to good prognosis in BLCA.

Supplementary Table 1: List of testis-biased genes. List of 1103 testis-biased genes together with absolute (FPKM) and proportional (%) expression levels in all three datasets (HBM, GTEx and HPA) used in this work.

See Supplementary File 1

Supplementary Table 2: List of putative CT genes. List of putative CT genes with percentage (Percent) of samples with expression and the corresponding S-score for all 15 tumor types used in this study.

See Supplementary File 2

Supplementary Table 3: CT gene products identified by mass spectrometry. List of CT gene products identified by our mass spectrometry analysis. For each CT gene, we provide the number of samples in which the respective protein is identified, the number of identified peptides and the total peptide intensity.

See Supplementary File 3

Supplementary Table 4: CT genes associated to prognosis. For all CT genes associated to either good or poor prognosis, absolute data regarding survival and CT expression is provided. Furthermore, statistical data is provided as pvalue and qvalue. Finally, correlation between expression and CD8+ counts is provided as a Pearson score (R2). Each spreadsheet corresponds to a tumor type.

See Supplementary File 4
